# Supplementary material for: A catalog of single nucleotide changes distinguishing modern humans from archaic hominins
Source: Sci Rep. 2019 Jun 11;9:8463. doi: 10.1038/s41598-019-44877-x (PMC6560109; doi:10.1038/s41598-019-44877-x)
Supplement: Supplementary file 1 — Supplementary Information [file 41598_2019_44877_MOESM1_ESM.pdf]

Supplementary Material for

A catalog of single nucleotide changes distinguishing modern humans from archaic hominins

Martin Kuhlwilm, Cedric Boeckx

This PDF includes:

Supplementary Discussion
Supplementary Figures
Supplementary Table legends
Supplementary References

Supplementary discussion

1. Cell division

Among the 15 fixed protein-coding changes identified here but absent from previous analyses (Pääbo 2014; Prüfer et al. 2014), some might also contribute to complex modifications of pathways in cell division: The AHR protein is involved in cell cycle regulation (Puga et al. 2002) and shows an excess of HF changes on the human lineage, the dynein DNHD1 might be recruited to the kinetochore (Bader et al. 2011) and is overexpressed in fetal brain (Safran et al. 2010), and the SSH2 protein (two fixed changes, one of which is first described here; and one on the archaic lineage) might interact with spindle assembly checkpoint proteins (Bailey et al. 2015). SHROOM4, which is associated to a mental retardation syndrome with delayed speech and aggressive behavior (Stocco dos Santos et al. 2003), may also be relevant (Yoder and Hildebrand 2007). Other proteins that carry two HHMCs are involved in mitosis, for example the spindle checkpoint regulator CHEK1 (Zachos et al. 2017), the Dynein Axonemal Heavy Chain 1 (encoded by *DNAH1*), the mitotic regulator AZI1 (*CEP131*) (St-Denis et al. 2017), the Cyclin D2 (*CCND2*) and the Protein Tyrosine Phosphatase Receptor Type C (*PTPRC*) (Melkerson-Watson et al. 1994). Other genes with HHMCs that could be part of the same functional network are *KIF26B* (Wojcik et al. 2018), *DCHS1* (Cappello et al. 2013; Klaus et al. 2019), *FOXM1* (Thiru et al. 2014) and *FMR1* (Callan et al. 2010), which carry a putative enrichment of HF changes, and *TOP2A* (Yoshida and Azuma 2016). The TOP2A protein shows the largest number of interactions (53) with other HHMC-carrying proteins, while CHEK1, KIF18A, KIF15 and PTPRC are among highly-interacting proteins with more than ten interactions, which suggests that these proteins might function as interaction hubs in modifications of the cell division complex. Furthermore, enrichment in cell-cycle related GO categories has been found for candidate regions for ancient positive selection (Racimo 2016), and *ANAPC10* has been highlighted, containing two potentially disruptive intronic changes that are fixed derived in modern humans and ancestral in both Neanderthals and Denisovans. This gene carries a total of 39 HF changes (11 of them fixed) specific to modern humans, but none for archaics.

On the archaic lineage, we find an AHMC in the *ASPM* gene, along with 24 other HF changes, but none in modern humans, resulting in an excess of archaic SNCs. The proteins ASPM and CIT, which carries an AHMC that is listed among the most disruptive non-synonymous derived SNCs in archaics (Table S31 in (Castellano et al. 2014)), are known to co-localize to the midbody ring during cytokinesis and regulate spindle orientation by affecting the dynamics of astral microtubules (Gai et al. 2016). These proteins regulate astral microtubules and thus the orientation of cell division in archaics, whereas in modern humans we find proteins regulating kinetochore microtubules, thus the timing of cell division. This difference could indicate two alternative ways of modulating cell division on the different lineages.

2. Cellular properties of neurons

The establishment of new connections requires protection, particularly as some of these connections reach long distance and are associated with enhanced activity following rewiring events, like for vocal motor neurons in songbirds (Pfenning et al. 2014). The gene *MAL*, which is implicated in myelin biogenesis and function, shows up in selective sweep regions (Racimo 2016;

Peyrégne et al. 2017) and is enriched for HF changes on the human lineage, while its orthologue *MAL2* carries a HHMC. A gene with HHMCs that is associated with the organization of the axon initial segment and nodes of Ranvier during early development is *NFASC* (Ango et al. 2004). The protein encoded by this gene is a L1 family immunoglobulin cell adhesion molecule, and we find that also the *L1CAM* gene carries an AHMC (Pollerberg et al. 2013). NFASC is also an interactor of DCX (Yap et al. 2012), which might have been under positive selection in humans (Peyrégne et al. 2017) and is enriched for HF SNCs on the human lineage, but carries an AHMC as well. At least three genes associated with the process and timing of myelination, *PTEN* (Harrington et al. 2010), *VCAN* (Dours-Zimmermann et al. 2009) and *NCMAP* (Ryu et al. 2008) are among genes with an excess of HF SNCs in modern humans. Other genes carrying HHMCs in our dataset associated with myelination include *SCAP* (Verheijen et al. 2009), *RB1CC1* (Menzies et al. 2015), *TENM4* (Hor et al. 2015), *CDKL1* (Hsu et al. 2011) and *ADSL* (Jurecka et al. 2012), and genes with an excess of changes on the human lineage with similar functions include *FBXW7* (Kearns et al. 2015), *KIFAP3* (Morfini et al. 2009), *PHLPP1* (Gunz et al. 2019), and *AMPH* (Butler et al. 1997). The AMPH protein interacts closely with the huntingtin protein HTT (which also carries a HHMC) and is involved in myelination processes (Huang et al. 2015).

Another interesting class that emerges from the set of genes is related to synaptic vesicle endocytosis, critical to sustain a high rate of synaptic transmission. We find a formal enrichment of genes with an excess of HF changes on the human compared to the archaic lineage with gene products located in the postsynaptic membrane and dendrites. *PACSIN1* (Widagdo et al. 2016) carries a HHMC, is among genes with an excess of HF changes, and has been highlighted as putatively under positive selection on the human lineage, along with other synaptic plasticity related genes such as *SIPA1L1* (Zhou et al. 2015; Racimo 2016; Peyrégne et al. 2017), *SH3GL2* (Arranz et al. 2015) and *STX1A* (Craig et al. 2015). Among genes harboring HHMCs and related to synaptic vesicle endocytosis, we find *LMNB2* (Razafsky et al. 2016) and *SV2C* (Janz and Südhof 1999). Finally, *SYT1*, which is critical for synaptic vesicle formation (Lee and Littleton 2015), carries a deleterious HHMC (Table 2).

Synaptic properties have been mentioned before in the context of human specific traits, for instance in postnatal brain development in humans, chimpanzees and macaques (Liu et al. 2012), with a focus on synaptogenesis and synaptic elimination in the prefrontal cortex. A period of high synaptic plasticity in humans has been related to a cluster of genes around a transcription factor encoded by the *MEF2A* gene. Even though this gene neither carries a protein-altering change nor shows a particular pattern in our analysis, any of the 26 HF SNCs it harbors on the modern human lineage could have had a functional impact not captured here. Apart from that, several of the genes with an excess of HF changes in modern humans do belong to this cluster: *CLSTN1, FBXW7, GABBR2, NRXN3, PTPRJ, PTPRN2, SLIT3*, and *STX1A*, three of which (*CLSTN1, FBXW7* and *STX1A*) are associated with signals of positive selection (Peyrégne et al. 2017). In addition, the above-mentioned AMPH interacts via CDKL5 (Sekiguchi et al. 2013) with HDAC4 (Trazzi et al. 2016). The latter exhibits an excess of HF changes in modern humans, and is known to repress the transcriptional activation of *MEF2A* (Miska et al. 1999). A putative signature of positive selection upstream of *MEF2A* (Somel et al. 2014) suggests that this may be part of a broader network which might be supported by our analysis. Finally, *ENTHD1/CACNA1I*, which contains a HHMC that can no longer be considered as fixed, but occurs at a very high frequency (>99.9%), lies in a selective sweep region (Peyrégne et al. 2017). The protein encoded by this gene is involved in synaptic vesicle endocytosis at nerve terminals (Ryan 2006) and is regulated by the *MEF2* gene family (Kornilov et al. 2016).

3. The brain growth trajectory

Changes in genes that influence microcephaly are found on both lineages: In archaics there are AHMCs in the microcephaly candidate genes *ASPM* (Tungadi et al. 2017) and *CIT* (Bianchi et al. 2017). The ASPM-katanin complex controls microtubule disassembly at spindle poles and misregulation of this process can lead to microcephaly (Jiang et al. 2017), which is of interest given the presence of a HHMC in *KATNA1* and a fixed non-coding change in *KATNB1*, while no such changes were observed in archaics (Yigit et al. 2016). Other genes associated with microcephaly that harbor non-synonymous SNCs are *CASC5* (two in humans, one in archaics) (Genin et al. 2012), *CDK5RAP2* (in humans), *MCPH1* (in archaics) (Arroyo et al. 2017), *ATRX* (one in humans and archaics each) (Ritchie et al. 2014), and *NHEJ1* (El Waly et al. 2015) (a deleterious one in humans, and one in archaics). Disease mutations in *SCAP* or *ADSL* have also been associated with microcephaly phenotypes as well (Suzuki et al. 2013; Jurecka et al. 2015), and Formin-2 (*FMN2*), which carries a deleterious regulatory change in modern humans, influences the development of the brain causing microcephaly in mice (Lian et al. 2016).

The SPAG5 protein, itself a microcephaly candidate gene (Boonsawat et al. 2019), carries three fixed HHMCs, has been claimed to interact with CDK5RAP2 (Kodani et al. 2015), is a direct target of PAX6 (Asami et al. 2011), via which it affects cell division orientation, and therefore is critical in the course of brain development. The *SPAG5* gene might be a particular example of specific consequences for a relevant SNC on the human lineage: One of the three fixed non-synonymous changes in the SPAG5 protein is a Proline-to-Serine substitution at position 43. This position is phosphorylated in humans (Dephoure et al. 2008) during the mitotic phase of the cell cycle, directly through the protein phosphatase 6 (PPP6C) at the Serine at this position (Rusin et al. 2015), with the effect of a modification of the duration of the metaphase. PPP6C regulates the mitotic spindle formation (Zeng et al. 2010), and the *PPP6C* gene itself carries five HF SNCs on the modern human lineage, one of which is a transcription factor binding site (for HNF4A/HNF4G), and only one SNC on the archaic lineage. This specific substitution in *SPAG5* seems likely to influence the duration of the metaphase through phosphorylation, as a molecular consequence of this HHMC.

Among macrocephaly-related genes with HHMCs in *RNF135* (Douglas et al. 2007), *CUL4B* (Tarpey et al. 2007) and *CCND2* (Mirzaa et al. 2014), the latter also shows a large number of HF changes on the human lineage, and the HHMC in *CUL4B* is inferred to be deleterious (Table 2). Other macrocephaly candidates such as *NFIX* (Klaassens et al. 2014), *NSD1* (Buxbaum et al. 2007) and *GLI3* (Jamsheer et al. 2012) have been claimed to have played an important role in shaping the distinctly modern human head (Gokhman et al. 2017) and show numerous SNCs in non-coding regions. *GLI3* might have been under positive selection (Peyrégne et al. 2017) and carries 20 HF SNCs on the human, but only one on the archaic lineage. Two of the very few genes hypothesized to regulate expansion and folding of the mammalian cerebral cortex by controlling radial glial cell number and fate, *TRNP1* (Stahl et al. 2013) and *TMEM14B* (Liu et al. 2017), exhibit HF 3'-UTR changes in modern humans, and *TRNP1* shows an excess of changes on the modern human lineage. The expression of these two genes in the outer subventricular zone might be important (Martínez-Martínez et al. 2016), since this is a critical region for complexification of neocortical growth in primates (Dehay et al. 2015), and for which an enriched activation of mTOR signaling has been reported (Nowakowski et al. 2017). In addition to other genes in the mTOR-pathway, such as *PTEN* (Li et al. 2017) or *CCND2*, two possibly interacting modulators (Cloutier et al. 2017) of the mTOR

signaling pathway stand out in our dataset: *ZNHIT2* with one deleterious SNC (Table 2) might have been under positive selection (Peyrégne et al. 2017), and *CCT6B* carries a deleterious change according to both SIFT and C-score. The transcription factor encoded by *RB1CC1* is essential for maintaining adult neuronal stem cells in the subventricular zone of the cerebral cortex (Wang et al. 2013). This gene carries a HHMC, a regulatory SNC that has been suggested to modify transcriptional activity (Weyer and Pääbo 2016), and a signature of positive selection (Prüfer et al. 2014). Lastly, it is noteworthy that *PALMD*, one of the 10 genes that do not contain any HF changes in archaics, but harbor an excess of HF changes in modern humans, has recently been shown to be critical for the morphology of basal progenitors, affecting their proliferative capacity, and thereby influencing neo-cortical expansion (Kalebic et al. 2019).

The number of HHMCs that putatively interact with proteins at the centrosome-cilium interface (Gupta et al. 2015) is more than expected using 1,000 random gene sets of a similar length distribution, for which 98.9% contain fewer genes with HHMCs. However, 99.9% of random sets also contain fewer genes with AHMCs, suggesting that differences between humans and archaics might lie in the particular genes rather than their numbers. On the archaic side, an enrichment of genes with AHMCs associated to “Corneal structure” may relate to archaic-specific changes in brain growth-trajectories since the size and position of the frontal and temporal lobes might affect eye and orbit morphology (Pereira-Pedro et al. 2017), and the macrocephaly-associated gene *RIN2* (Basel-Vanagaite et al. 2009) carries an AHMC.

4. The impact on cognition

It has long been hypothesized that language and its neurological foundation were important for the evolution of humans and uniquely human traits, closely related to hypotheses on the evolution of cognition and behavior. It is noteworthy that among traits associated with cognitive functions such as language or theory of mind, the timing of myelination appears to be a good predictor of computational abilities (Skeide and Friederici 2016; Grosse Wiesmann et al. 2017). We suggest that some genes with changes on the human lineage might have contributed more specifically to cognition-related changes, although we admit that the contribution of single SNCs to these functions is less straightforward than their contribution to molecular mechanisms, since disease mutations in many genes may have disruptive effects on cognitive abilities. The basal ganglia are a brain region where *FOXP2* expression is critical for the establishment and maintenance of language-related functions (Vargha-Khadem et al. 2005; Enard et al. 2009), and several genes carrying HHMCs have been described previously as important for basal ganglia functions (also, Neanderthal alleles influence the expression of *UBR4* in basal ganglia, and reduce globularity (Gunz et al. 2019)). The HTT protein has long been implicated in the development of Huntington’s disease, which is associated with corticostriatal dysfunction, and is known to interact with FOXP2 (Hachigian et al. 2017). Mutations in *SLITRK1*, which might have been under positive selection (Peyrégne et al. 2017), have been linked to Tourette's syndrome, a disorder characterized by vocal and motor tics, resulting from a dysfunction in the corticostriatal-thalamocortical circuits (Abelson et al. 2005). NOVA1 regulates RNA splicing and metabolism in a specific subset of developing neurons, particularly in the striatum (Jelen et al. 2010). As pointed out above, *NOVA1* is an interactor of *ELAVL4*, which belongs to a family of genes known to promote the production of deep layer *FOXP2*-expressing neurons (Konopka et al. 2012; Alsiö et al. 2013; Popovitchenko et al. 2016), and part of a neural network-related cluster that has been highlighted as putatively under

positive selection in humans (Zhou et al. 2015). Within this network, α-synuclein (encoded by *SCNA*) might serve as a hub and is specifically expressed in brain regions important for vocal learning regions in songbirds (Pfenning et al. 2014). *SCNA* and *SV2C*, which carries a HHMC, are involved in the regulation of dopamine release, with *SV2C* expression being disrupted in *SCNA*-deficient mice and in humans with Parkinson's disease (Dunn et al. 2017). Genes in the cluster of selection signals (Zhou et al. 2015) are implicated in the pathogenesis of Alzheimer's disease, which (together with Huntingon's and Parkinson's diseases) is linked to a *FOXP2*-driven network (Oswald et al. 2017). Some introgressed archaic alleles are downregulated in specific brain regions (McCoy et al. 2017), especially pronounced in the cerebellum and basal ganglia. One notable example is *NTRK2*, which shows an excess of HF changes on the human lineage and a signature of positive selection (Peyrégne et al. 2017), and is also a FOXP2 target (Vernes et al. 2011), a connection which has been highlighted for the vocal learning circuit in birds (Hilliard et al. 2012). Other genes harboring HHMCs such as *ENTHD1* (Kornilov et al. 2016) and *STARD9* (Chen et al. 2017), as well as genes in introgression deserts (Vernot et al. 2016), have been associated with language deficits. It may indeed have taken a complex composite of changes to make our brain fully language-ready (Boeckx and Benítez-Burraco 2014), where not all changes needed to reach fixation due to pleiotropy of their functions.

Two genes linked to Alzheimer's are *PTEN* (Ferrarelli 2016), and *RB1CC1* (Chano et al. 2007). Among genes with deleterious HHMCs, *SLC6A15* has been associated to emotional processing in the brain (Choi et al. 2016), and may be part of modifications in glutamatergic transmission (Santarelli et al. 2016), a category found in selective sweep regions (Theofanopoulou et al. 2017). *GPR153*, which carries one HHMC and two AHMCs, influences behavioral traits like decision making in rats, and is associated with various neuropsychiatric disorders in humans (Sreedharan et al. 2011). For the Adenylosuccinate Lyase (*ADSL*) the ancestral Neanderthal-like allele has not been observed in 1,000s of modern human genomes. This gene has been associated to autism (Fon et al. 1995), is part of behavioral traits like "aggressive behavior" which have been found to be enriched on the human lineage (Castellano et al. 2014), and several studies detected a signal of positive selection in modern humans (Racimo et al. 2014; Racimo 2016; Peyrégne et al. 2017). These observations make *ADSL* a strong candidate for human-specific features, particularly in light of the fact that the relevant HHMC is located in a region that is highly conserved and lies close to the most common disease mutation leading to severe adenylosuccinase deficiency (Racimo 2016). Other relevant genes, similar to *ADSL* in carrying a fixed HHMC and being frequently found in selective sweep screens, are *NCOA6*, which might be related to autism as well (Takata et al. 2018), and *SCAP*. Downregulation of the cholesterol sensor encoded by this gene has been shown to cause microcephaly, impaired synaptic transmission and altered cognitive function in mice (Suzuki et al. 2013). We want to emphasize that the networks presented in the previous sections influencing brain growth and neural wiring are likely to impact cognitive functions, since disruptions in these networks would impair the healthy human brain. Furthermore, we find an enrichment of AHMCs in genes associated to Parkinson's disease and "Attention deficit hyperactivity disorder and conduct disorder", suggesting that changes may have taken place in related networks on the archaic lineage as well.

Supplementary Figures

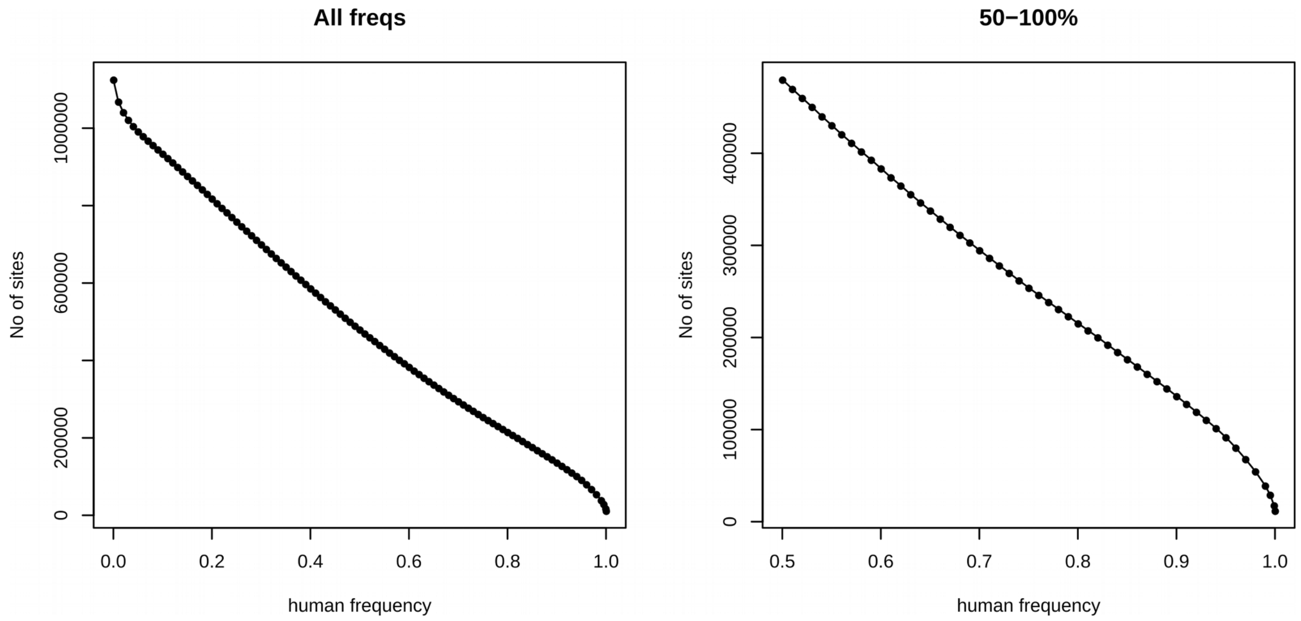

Figure S1: Total number of differences between modern humans and archaics, as considered by increasing frequency cutoffs. X-axis, allele frequency in humans. Right panel: Zoom into frequencies larger than 50% in modern humans. Above 90% frequency, the number drops more more sharply.

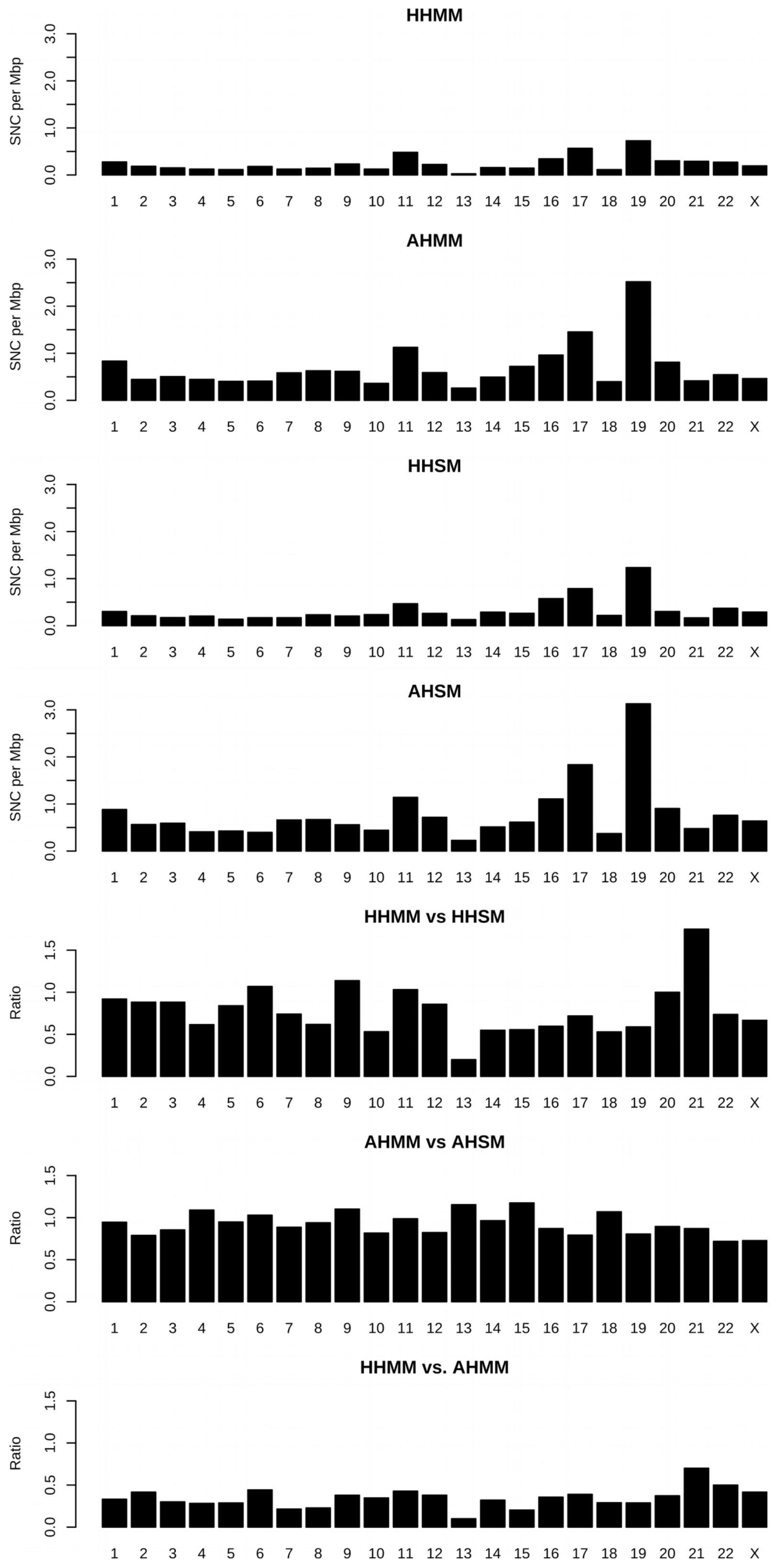

Figure S2: Distribution of missense and non-synonymous HF SNCs across chromosomes. From top top bottom: Number of HHMCs per million base pairs (Mbp), number of AHMCs per Mbp, number of synonymous HF changes on the human lineage per Mbp, number of synonymous HF changes on the archaic lineage per Mbp, ratio of non-synonymous to synonymous HF changes on the human lineage, ratio of non-synonymous to synonymous HF changes on the archaic lineage, ratio of HHMCs to AHMCs.

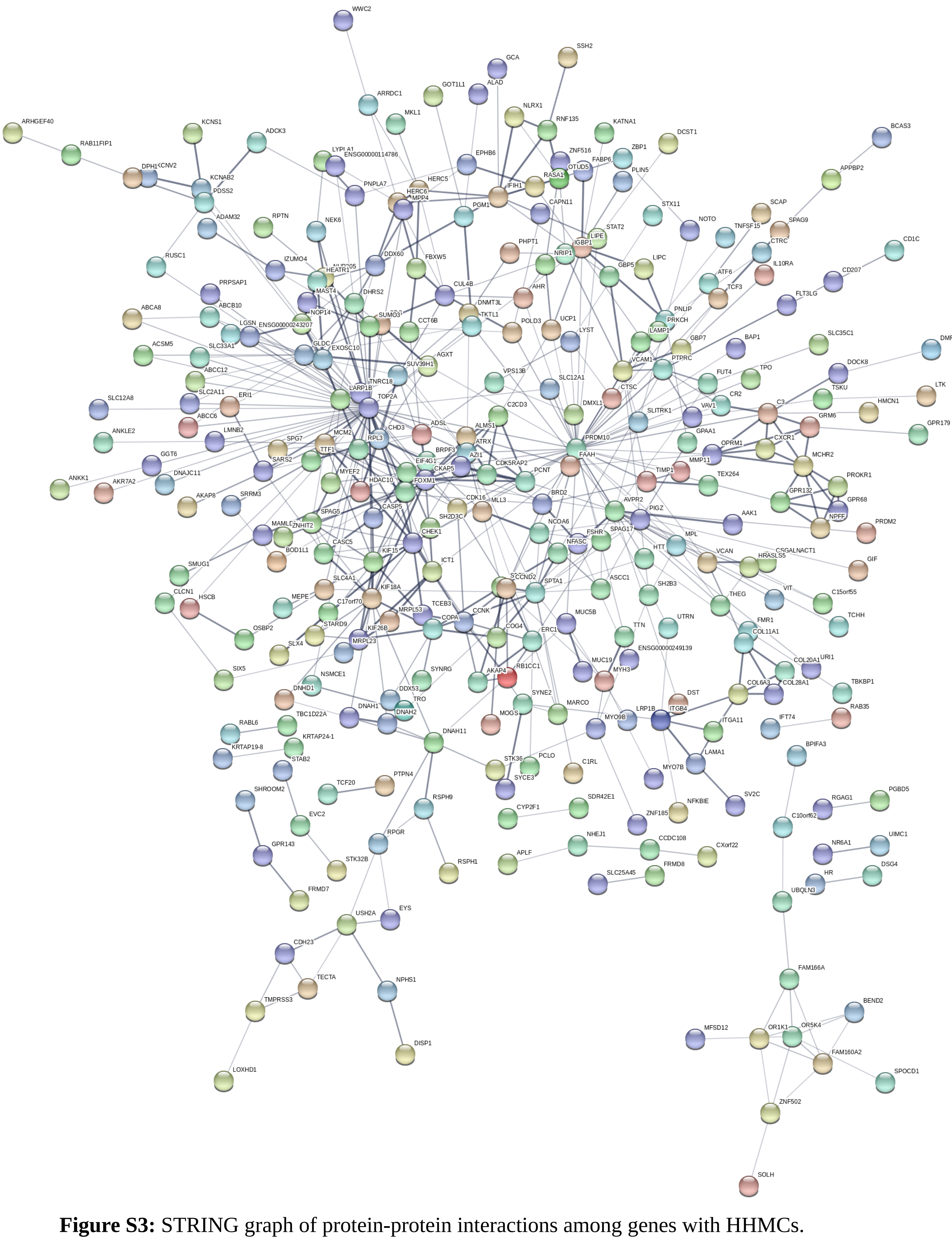

Figure S3: STRING graph of protein-protein interactions among genes with HHMCs.

Supplementary Table legends

Table S1: List of HHMCs and genomic features

Table S2: List of low-confidence HHMCs and genomic features

Table S3: List of AHMCs and genomic features

Table S4: List of low-confidence AHMCs and genomic features

Table S5: Top 5% of genes by HF SNC density on the modern human and archaic lineages, and top 10% of genes by relative excess of HF SNCs on one lineage over the other.

Table S6: GO enrichment for genes with relative excess of HF SNCs on the human over the archaic lineage

Table S7: GWAS enrichment for genes with HHMCs or AHMCs

Table S8: Number of interactions among genes with HHMCs or AHMCs

Table S9: Genes with HHMCs that are transcription factors, or at the centrosome interface

Table S10: Enrichment in developing brain zones for genes with HHMCs or AHMCs, proportion of random gene sets with larger overlap (Methods).

Supplementary References

Abelson JF, Kwan KY, O'Roak BJ, Baek DY, Stillman AA, Morgan TM, Mathews CA, Pauls DL, Rašin M-R, Gunel M, et al. 2005. Sequence Variants in SLITRK1 Are Associated with Tourette's Syndrome. Science (80-.). [Internet] 310:317 LP-320. Available from: http://science.sciencemag.org/content/310/5746/317.abstract

Alsiö JM, Tarchini B, Cayouette M, Livesey FJ. 2013. Ikaros promotes early-born neuronal fates in the cerebral cortex. Proc. Natl. Acad. Sci. [Internet] 110:E716–E725. Available from: http://www.pnas.org/content/110/8/E716.abstract

Ango F, di Cristo G, Higashiyama H, Bennett V, Wu P, Huang ZJ. 2004. Ankyrin-Based Subcellular Gradient of Neurofascin, an Immunoglobulin Family Protein, Directs GABAergic Innervation at Purkinje Axon Initial Segment. Cell [Internet] 119:257–272. Available from: http://www.sciencedirect.com/science/article/pii/S009286740400947X

Arranz AM, Delbroek L, Van Kolen K, Guimarães MR, Mandemakers W, Daneels G, Matta S, Calafate S, Shaban H, Baatsen P, et al. 2015. LRRK2 functions in synaptic vesicle endocytosis through a kinase-dependent mechanism. J. Cell Sci. [Internet] 128:541 LP-552. Available from: http://jcs.biologists.org/content/128/3/541.abstract

Arroyo M, Kuriyama R, Trimborn M, Keifenheim D, Cañuelo A, Sánchez A, Clarke DJ, Marchal JA. 2017. MCPH1, mutated in primary microcephaly, is required for efficient chromosome alignment during mitosis. Sci. Rep. [Internet] 7:13019. Available from: https://doi.org/10.1038/s41598-017-12793-7

Asami M, Pilz GA, Ninkovic J, Godinho L, Schroeder T, Huttner WB, Götz M. 2011. The role of Pax6 in regulating the orientation and mode of cell division of progenitors in the mouse cerebral cortex. Development [Internet] 138:5067 LP-5078. Available from: http://dev.biologists.org/content/138/23/5067.abstract

Bader JR, Kasuboski JM, Winding M, Vaughan PS, Hinchcliffe EH, Vaughan KT. 2011. Polo-like Kinase1 Is Required for Recruitment of Dynein to Kinetochores during Mitosis. J. Biol. Chem. [Internet] 286:20769–20777. Available from: http://www.jbc.org/content/286/23/20769.abstract

Bailey ML, Singh T, Mero P, Moffat J, Hieter P. 2015. Dependence of Human Colorectal Cells Lacking the FBW7 Tumor Suppressor on the Spindle Assembly Checkpoint. Genetics [Internet] 201:885 LP-895. Available from: http://www.genetics.org/content/201/3/885.abstract

Basel-Vanagaite L, Sarig O, Hershkovitz D, Fuchs-Telem D, Rapaport D, Gat A, Isman G, Shirazi I, Shohat M, Enk CD, et al. 2009. RIN2 Deficiency Results in Macrocephaly, Alopecia, Cutis Laxa, and Scoliosis: MACS Syndrome. Am. J. Hum. Genet. [Internet] 85:254–263. Available from: http://dx.doi.org/10.1016/j.ajhg.2009.07.001

Bianchi FT, Tocco C, Pallavicini G, Liu Y, Vernì F, Merigliano C, Bonaccorsi S, El-Assawy N, Priano L, Gai M, et al. 2017. Citron Kinase Deficiency Leads to Chromosomal Instability and TP53-Sensitive Microcephaly. Cell Rep. [Internet] 18:1674–1686. Available from: http://dx.doi.org/10.1016/j.celrep.2017.01.054

Boeckx C, Benítez-Burraco A. 2014. Globularity and language-readiness: generating new predictions by expanding the set of genes of interest . Front. Psychol. [Internet] 5:1324. Available from: https://www.frontiersin.org/article/10.3389/fpsyg.2014.01324

Boonsawat P, Joset P, Steindl K, Oneda B, Gogoll L, Azzarello-Burri S, Sheth F, Datar C, Verma IC, Puri RD, et al. 2019. Elucidation of the phenotypic spectrum and genetic landscape in primary and secondary microcephaly. Genet. Med. [Internet]. Available from: https://doi.org/10.1038/s41436-019-0464-7

Butler MH, David C, Ochoa G-C, Freyberg Z, Daniell L, Grabs D, Cremona O, Camilli P De. 1997. Amphiphysin II (SH3P9; BIN1), a Member of the Amphiphysin/Rvs Family, Is Concentrated in the Cortical Cytomatrix of Axon

Initial Segments and Nodes of Ranvier in Brain and around T Tubules in Skeletal Muscle. J. Cell Biol. [Internet] 137:1355–1367. Available from: http://www.ncbi.nlm.nih.gov/pmc/articles/PMC2132527/

Buxbaum JD, Cai G, Nygren G, Chaste P, Delorme R, Goldsmith J, Råstam M, Silverman JM, Hollander E, Gillberg C, et al. 2007. Mutation analysis of the NSD1 gene in patients with autism spectrum disorders and macrocephaly. BMC Med. Genet. [Internet] 8:68. Available from: https://doi.org/10.1186/1471-2350-8-68

Callan MA, Cabernard C, Heck J, Luois S, Doe CQ, Zarnescu DC. 2010. Fragile X protein controls neural stem cell proliferation in the Drosophila brain . Hum. Mol. Genet. [Internet] 19:3068–3079. Available from: http://dx.doi.org/10.1093/hmg/ddq213

Cappello S, Gray MJ, Badouel C, Lange S, Einsiedler M, Srour M, Chitayat D, Hamdan FF, Jenkins ZA, Morgan T, et al. 2013. Mutations in genes encoding the cadherin receptor-ligand pair DCHS1 and FAT4 disrupt cerebral cortical development. Nat. Genet. [Internet] 45:1300. Available from: http://dx.doi.org/10.1038/ng.2765

Castellano S, Parra G, Sánchez-Quinto FA, Racimo F, Kuhlwilm M, Kircher M, Sawyer S, Fu Q, Heinze A, Nickel B, et al. 2014. Patterns of coding variation in the complete exomes of three Neandertals. Proc. Natl. Acad. Sci. U. S. A. [Internet] 111:6666–6671. Available from: http://www.pnas.org/content/111/18/6666

Chano T, Okabe H, Hulette CM. 2007. RB1CC1 insufficiency causes neuronal atrophy through mTOR signaling alteration and involved in the pathology of Alzheimer’s diseases. Brain Res. [Internet] 1168:97–105. Available from: http://www.sciencedirect.com/science/article/pii/S0006899307016423

Chen XS, Reader RH, Hoischen A, Veltman JA, Simpson NH, Francks C, Newbury DF, Fisher SE. 2017. Next-generation DNA sequencing identifies novel gene variants and pathways involved in specific language impairment. Sci. Rep. [Internet] 7:46105. Available from: http://dx.doi.org/10.1038/srep46105

Choi S, Han K-M, Kang J, Won E, Chang HS, Tae WS, Son KR, Kim S-J, Lee M-S, Ham B-J. 2016. Effects of a Polymorphism of the Neuronal Amino Acid Transporter SLC6A15 Gene on Structural Integrity of White Matter Tracts in Major Depressive Disorder. PLoS One [Internet] 11:e0164301. Available from: https://doi.org/10.1371/journal.pone.0164301

Cloutier P, Poitras C, Durand M, Hekmat O, Fiola-Masson É, Bouchard A, Faubert D, Chabot B, Coulombe B. 2017. R2TP/Prefoldin-like component RUVBL1/RUVBL2 directly interacts with ZNHIT2 to regulate assembly of U5 small nuclear ribonucleoprotein. Nat. Commun. [Internet] 8:15615. Available from: http://dx.doi.org/10.1038/ncomms15615

Craig TJ, Anderson D, Evans AJ, Girach F, Henley JM. 2015. SUMOylation of Syntaxin1A regulates presynaptic endocytosis. Sci. Rep. [Internet] 5:17669. Available from: http://dx.doi.org/10.1038/srep17669

Dehay C, Kennedy H, Kosik KS. 2015. The Outer Subventricular Zone and Primate-Specific Cortical Complexification. Neuron [Internet] 85:683–694. Available from: http://dx.doi.org/10.1016/j.neuron.2014.12.060

Dephoure N, Zhou C, Villén J, Beausoleil SA, Bakalarski CE, Elledge SJ, Gygi SP. 2008. A quantitative atlas of mitotic phosphorylation. Proc. Natl. Acad. Sci. [Internet] 105:10762–10767. Available from: http://www.pnas.org/content/105/31/10762.abstract

Douglas J, Cilliers D, Coleman K, Tatton-Brown K, Barker K, Bernhard B, Burn J, Huson S, Josifova D, Lacombe D, et al. 2007. Mutations in RNF135, a gene within the NF1 microdeletion region, cause phenotypic abnormalities including overgrowth. Nat. Genet. [Internet] 39:963. Available from: http://dx.doi.org/10.1038/ng2083

Dours-Zimmermann MT, Maurer K, Rauch U, Stoffel W, Fässler R, Zimmermann DR. 2009. Versican V2 Assembles the Extracellular Matrix Surrounding the Nodes of Ranvier in the CNS. J. Neurosci. [Internet] 29:7731 LP-7742. Available from: http://www.jneurosci.org/content/29/24/7731.abstract

Dunn AR, Stout KA, Ozawa M, Lohr KM, Hoffman CA, Bernstein AI, Li Y, Wang M, Sgobio C, Sastry N, et al. 2017. Synaptic vesicle glycoprotein 2C (SV2C) modulates dopamine release and is disrupted in Parkinson disease. Proc. Natl. Acad. Sci. [Internet] 114:E2253–E2262. Available from: http://www.pnas.org/content/114/11/E2253.abstract

Enard W, Gehre S, Hammerschmidt K, Hölter SM, Blass T, Somel M, Brückner MK, Schreiweis C, Winter C, Sohr R, et al. 2009. A Humanized Version of Foxp2 Affects Cortico-Basal Ganglia Circuits in Mice. Cell [Internet] 137:961–971. Available from: http://linkinghub.elsevier.com/retrieve/pii/S009286740900378X

Ferrarelli LK. 2016. PTEN contributes to Alzheimer's disease. Sci. Signal. [Internet] 9:ec45 LP-ec45. Available from: http://stke.sciencemag.org/content/9/417/ec45.abstract

Fon EA, Sarrazin J, Meunier C, Alarcia J, Shevell MI, Philippe A, Leboyer M, Rouleau GA. 1995. Adenylosuccinate lyase (ADSL) and infantile autism: Absence of previously reported point mutation. Am. J. Med. Genet. [Internet] 60:554–557. Available from: http://doi.wiley.com/10.1002/ajmg.1320600614

Gai M, Bianchi FT, Vagnoni C, Vernì F, Bonaccorsi S, Pasquero S, Berto GE, Sgrò F, Chiotto AMA, Annaratone L, et al. 2016. ASPM and CITK regulate spindle orientation by affecting the dynamics of astral microtubules. EMBO Rep. [Internet] 17:1396–1409. Available from: http://dx.doi.org/10.15252/embr.201541823

Genin A, Desir J, Lambert N, Biervliet M, Van Der Aa N, Pierquin G, Killian A, Tosi M, Urbina M, Lefort A, et al. 2012. Kinetochore KMN network gene CASC5 mutated in primary microcephaly. Hum. Mol. Genet. [Internet] 21:5306–5317. Available from: http://dx.doi.org/10.1093/hmg/dds386

Gokhman D, Agranat-Tamir L, Housman G, Garcia-Perez R, Nissim-Rafinia M, Mallick S, Nieves-Colón M, Li H, Alpaslan-Roodenberg S, Novak M, et al. 2017. Extensive Regulatory Changes in Genes Affecting Vocal and Facial Anatomy Separate Modern from Archaic Humans. bioRxiv [Internet]. Available from: http://biorxiv.org/content/early/2017/10/03/106955.abstract

Grosse Wiesmann C, Schreiber J, Singer T, Steinbeis N, Friederici AD. 2017. White matter maturation is associated with the emergence of Theory of Mind in early childhood. Nat. Commun. [Internet] 8:14692. Available from: http://dx.doi.org/10.1038/ncomms14692

Gunz P, Tilot AK, Wittfeld K, Teumer A, Shapland CY, van Erp TGM, Dannemann M, Vernot B, Neubauer S, Guadalupe T, et al. 2019. Neandertal Introgression Sheds Light on Modern Human Endocranial Globularity. Curr. Biol. [Internet] 29:120–127.e5. Available from: https://doi.org/10.1016/j.cub.2018.10.065

Gupta GD, Coyaud É, Gonçalves J, Mojarad BA, Liu Y, Wu Q, Gheiratmand L, Comartin D, Tkach JM, Cheung SWT, et al. 2015. A Dynamic Protein Interaction Landscape of the Human Centrosome-Cilium Interface. Cell [Internet] 163:1484–1499. Available from: http://dx.doi.org/10.1016/j.cell.2015.10.065

Hachigian LJ, Carmona V, Fenster RJ, Kulicke R, Heilbut A, Sittler A, Pereira de Almeida L, Mesirov JP, Gao F, Kolaczyk ED, et al. 2017. Control of Huntington’s Disease-Associated Phenotypes by the Striatum-Enriched Transcription Factor Foxp2. Cell Rep. [Internet] 21:2688–2695. Available from: http://dx.doi.org/10.1016/j.celrep.2017.11.018

Harrington EP, Zhao C, Fancy SPJ, Kaing S, Franklin RJM, Rowitch DH. 2010. Oligodendrocyte PTEN is required for myelin and axonal integrity, not remyelination. Ann. Neurol. [Internet] 68:703–716. Available from: http://doi.wiley.com/10.1002/ana.22090

Hilliard AT, Miller JE, Fraley ER, Horvath S, White SA. 2012. Molecular Microcircuitry Underlies Functional Specification in a Basal Ganglia Circuit Dedicated to Vocal Learning. Neuron [Internet] 73:537–552. Available from: http://dx.doi.org/10.1016/j.neuron.2012.01.005

Hor H, Francescatto L, Bartesaghi L, Ortega-Cubero S, Kousi M, Lorenzo-Betancor O, Jiménez-Jiménez FJ, Gironell A, Clarimón J, Drechsel O, et al. 2015. Missense mutations in TENM4, a regulator of axon guidance and central myelination, cause essential tremor. Hum. Mol. Genet. [Internet] 24:5677–5686. Available from: http://dx.doi.org/10.1093/hmg/ddv281

Hsu L-S, Liang C-J, Tseng C-Y, Yeh C-W, Tsai J-N. 2011. Zebrafish Cyclin-Dependent Protein Kinase–Like 1 (zcdkl1): Identification and Functional Characterization. Int. J. Mol. Sci. [Internet] 12:3606–3617. Available from: http://www.mdpi.com/1422-0067/12/6/3606/

Huang B, Wei W, Wang G, Gaertig MA, Feng Y, Wang W, Li X-J, Li S. 2015. Mutant Huntingtin Downregulates Myelin Regulatory Factor-Mediated Myelin Gene Expression and Affects Mature Oligodendrocytes. Neuron [Internet] 85:1212–1226. Available from: http://dx.doi.org/10.1016/j.neuron.2015.02.026

Jamsheer A, Sowińska A, Trzeciak T, Jamsheer-Bratkowska M, Geppert A, Latos-Bieleńska A. 2012. Expanded mutational spectrum of the GLI3 gene substantiates genotype–phenotype correlations. J. Appl. Genet. [Internet] 53:415–422. Available from: https://doi.org/10.1007/s13353-012-0109-x

Janz R, Südhof TC. 1999. SV2C is a synaptic vesicle protein with an unusually restricted localization: anatomy of a synaptic vesicle protein family. Neuroscience [Internet] 94:1279–1290. Available from: http://www.sciencedirect.com/science/article/pii/S030645229900370X

Jelen N, Ule J, Živin M. 2010. Cholinergic regulation of striatal Nova mRNAs. Neuroscience [Internet] 169:619–627. Available from: http://www.sciencedirect.com/science/article/pii/S0306452210007128

Jiang K, Rezabkova L, Hua S, Liu Q, Capitani G, Altelaar AFM, Heck AJR, Kammerer RA, Steinmetz MO, Akhmanova A. 2017. Microtubule minus-end regulation at spindle poles by an ASPM–katanin complex. Nat. Cell Biol. [Internet] 19:480. Available from: http://dx.doi.org/10.1038/ncb3511

Jurecka A, Jurkiewicz E, Tylki-Szymanska A. 2012. Magnetic resonance imaging of the brain in adenylosuccinate lyase deficiency: a report of seven cases and a review of the literature. Eur. J. Pediatr. [Internet] 171:131–138. Available from: https://doi.org/10.1007/s00431-011-1503-9

Jurecka A, Zikanova M, Kmoch S, Tylki-Szymańska A. 2015. Adenylosuccinate lyase deficiency. J. Inherit. Metab. Dis. [Internet] 38:231–242. Available from: https://doi.org/10.1007/s10545-014-9755-y

Kalebic N, Gilardi C, Stepien B, Wilsch-Bräuninger M, Long KR, Namba T, Florio M, Langen B, Lombardot B, Shevchenko A, et al. 2019. Neocortical Expansion Due to Increased Proliferation of Basal Progenitors Is Linked to Changes in Their Morphology. Cell Stem Cell [Internet] 24:535–550.e9. Available from: https://doi.org/10.1016/j.stem.2019.02.017

Kearns CA, Ravanelli AM, Cooper K, Appel B. 2015. Fbxw7 Limits Myelination by Inhibiting mTOR Signaling. J. Neurosci. [Internet] 35:14861 LP-14871. Available from: http://www.jneurosci.org/content/35/44/14861.abstract

Klaassens M, Morrogh D, Rosser EM, Jaffer F, Vreeburg M, Bok LA, Segboer T, van Belzen M, Quinlivan RM, Kumar A, et al. 2014. Malan syndrome: Sotos-like overgrowth with de novo NFIX sequence variants and deletions in six new patients and a review of the literature. Eur. J. Hum. Genet. [Internet] 23:610. Available from: http://dx.doi.org/10.1038/ejhg.2014.162

Klaus J, Kanton S, Kyrousi C, Ayo-Martin AC, Di Giaimo R, Riesenberg S, O'Neill AC, Camp JG, Tocco C, Santel M, et al. 2019. Altered neuronal migratory trajectories in human cerebral organoids derived from individuals with neuronal heterotopia. Nat. Med. [Internet] 25:561–568. Available from: https://doi.org/10.1038/s41591-019-0371-0

Kodani A, Yu TW, Johnson JR, Jayaraman D, Johnson TL, Al-Gazali L, Sztriha L, Partlow JN, Kim H, Krup AL, et al. 2015. Centriolar satellites assemble centrosomal microcephaly proteins to recruit CDK2 and promote centriole duplication.Nelson WJ, editor. Elife [Internet] 4:e07519. Available from: https://doi.org/10.7554/eLife.07519

Konopka G, Friedrich T, Davis-Turak J, Winden K, Oldham MC, Gao F, Chen L, Wang G-Z, Luo R, Preuss TM, et al. 2012. Human-Specific Transcriptional Networks in the Brain. Neuron [Internet] 75:601–617. Available from: http://dx.doi.org/10.1016/j.neuron.2012.05.034

Kornilov SA, Rakhlin N, Koposov R, Lee M, Yrigollen C, Caglayan AO, Magnuson JS, Mane S, Chang JT, Grigorenko EL. 2016. Genome-Wide Association and Exome Sequencing Study of Language Disorder in an Isolated Population. Pediatrics [Internet]. Available from: http://pediatrics.aappublications.org/content/early/2016/03/24/peds.2015-2469.abstract

Lee J, Littleton JT. 2015. Transmembrane tethering of synaptotagmin to synaptic vesicles controls multiple modes of neurotransmitter release. Proc. Natl. Acad. Sci. [Internet] 112:3793–3798. Available from: http://www.pnas.org/content/112/12/3793.abstract

Li Y, Muffat J, Omer A, Bosch I, Lancaster MA, Sur M, Gehrke L, Knoblich JA, Jaenisch R. 2017. Induction of Expansion and Folding in Human Cerebral Organoids. Cell Stem Cell [Internet] 20:385–396.e3. Available from: http://dx.doi.org/10.1016/j.stem.2016.11.017

Lian G, Dettenhofer M, Lu J, Downing M, Chenn A, Wong T, Sheen V. 2016. Filamin A- and formin 2-dependent endocytosis regulates proliferation via the canonical Wnt pathway. Development [Internet] 143:4509 LP-4520. Available from: http://dev.biologists.org/content/143/23/4509.abstract

Liu J, Liu W, Yang L, Wu Q, Zhang H, Fang A, Li L, Xu X, Sun L, Zhang J, et al. 2017. The Primate-Specific Gene TMEM14B Marks Outer Radial Glia Cells and Promotes Cortical Expansion and Folding. Cell Stem Cell [Internet] 21:635–649.e8. Available from: http://dx.doi.org/10.1016/j.stem.2017.08.013

Liu X, Somel M, Tang L, Yan Z, Jiang X, Guo S, Yuan Y, He L, Oleksiak A, Zhang Y, et al. 2012. Extension of cortical synaptic development distinguishes humans from chimpanzees and macaques. Genome Res. [Internet] 22:611–622. Available from: http://www.ncbi.nlm.nih.gov/pmc/articles/PMC3317144/

Martínez-Martínez MÁ, De Juan Romero C, Fernández V, Cárdenas A, Götz M, Borrell V. 2016. A restricted period for formation of outer subventricular zone defined by Cdh1 and Trnp1 levels. Nat. Commun. [Internet] 7:11812. Available from: http://dx.doi.org/10.1038/ncomms11812

McCoy RC, Wakefield J, Akey JM. 2017. Impacts of Neanderthal-Introgressed Sequences on the Landscape of Human Gene Expression. Cell [Internet] 168:916–927.e12. Available from: http://dx.doi.org/10.1016/j.cell.2017.01.038

Melkerson-Watson LJ, Waldmann ME, Gunter AD, Zaroukian MH, Esselman WJ. 1994. Elevation of lymphocyte CD45 protein tyrosine phosphatase activity during mitosis. J. Immunol. [Internet] 153:2004 LP-2013. Available from: http://www.jimmunol.org/content/153/5/2004.abstract

Menzies FM, Fleming A, Rubinsztein DC. 2015. Compromised autophagy and neurodegenerative diseases. Nat. Rev. Neurosci. [Internet] 16:345. Available from: http://dx.doi.org/10.1038/nrn3961

Mirzaa GM, Parry DA, Fry AE, Giamanco KA, Schwartzentruber J, Vanstone M, Logan C V, Roberts N, Johnson CA, Singh S, et al. 2014. De novo CCND2 mutations leading to stabilization of cyclin D2 cause megalencephaly-polymicrogyria-polydactyly-hydrocephalus syndrome. Nat. Genet. [Internet] 46:510. Available from: http://dx.doi.org/10.1038/ng.2948

Miska EA, Karlsson C, Langley E, Nielsen SJ, Pines J, Kouzarides T. 1999. HDAC4 deacetylase associates with and represses the MEF2 transcription factor. EMBO J. [Internet] 18:5099 LP-5107. Available from: http://emboj.embopress.org/content/18/18/5099.abstract

Morfini GA, Burns M, Binder LI, Kanaan NM, LaPointe N, Bosco DA, Brown RH, Brown H, Tiwari A, Hayward L, et al. 2009. Axonal Transport Defects in Neurodegenerative Diseases. J. Neurosci. [Internet] 29:12776 LP-12786. Available from: http://www.jneurosci.org/content/29/41/12776.abstract

Nowakowski TJ, Bhaduri A, Pollen AA, Alvarado B, Mostajo-Radji MA, Di Lullo E, Haeussler M, Sandoval-Espinosa C, Liu SJ, Velmeshev D, et al. 2017. Spatiotemporal gene expression trajectories reveal developmental hierarchies of the human cortex. Science (80-.). [Internet] 358:1318 LP-1323. Available from: http://science.sciencemag.org/content/358/6368/1318.abstract

Oswald F, Klöble P, Ruland A, Rosenkranz D, Hinz B, Butter F, Ramljak S, Zechner U, Herlyn H. 2017. The FOXP2-Driven Network in Developmental Disorders and Neurodegeneration . Front. Cell. Neurosci. [Internet] 11:212. Available from: https://www.frontiersin.org/article/10.3389/fncel.2017.00212

Pääbo S. 2014. The Human Condition—A Molecular Approach. Cell [Internet] 157:216–226. Available from: http://www.sciencedirect.com/science/article/pii/S009286741301605X

Pereira-Pedro AS, Masters M, Bruner E. 2017. Shape analysis of spatial relationships between orbito-ocular and endocranial structures in modern humans and fossil hominids. J. Anat. [Internet] 231:947–960. Available from: http://doi.wiley.com/10.1111/joa.12693

Peyrégne S, Boyle MJ, Dannemann M, Prüfer K. 2017. Detecting ancient positive selection in humans using extended lineage sorting. Genome Res. [Internet] 27:1563–1572. Available from: http://genome.cshlp.org/content/27/9/1563.abstract

Pfenning AR, Hara E, Whitney O, Rivas M V, Wang R, Roulhac PL, Howard JT, Wirthlin M, Lovell P V, Ganapathy G, et al. 2014. Convergent transcriptional specializations in the brains of humans and song-learning birds. Science (80-.). [Internet] 346. Available from: http://science.sciencemag.org/content/346/6215/1256846.abstract

Pollerberg GE, Thelen K, Theiss MO, Hochlehnert BC. 2013. The role of cell adhesion molecules for navigating axons: Density matters. Mech. Dev. [Internet] 130:359–372. Available from: http://www.sciencedirect.com/science/article/pii/S092547731200113X

Popovitchenko T, Thompson K, Viljetic B, Jiao X, Kontonyiannis DL, Kiledjian M, Hart RP, Rasin MR. 2016. The RNA binding protein HuR determines the differential translation of autism-associated FoxP subfamily members in the developing neocortex. Sci. Rep. [Internet] 6:28998. Available from: http://dx.doi.org/10.1038/srep28998

Prüfer K, Racimo F, Patterson N, Jay F, Sankararaman S, Sawyer S, Heinze A, Renaud G, Sudmant PHPH, de Filippo C, et al. 2014. The complete genome sequence of a Neanderthal from the Altai Mountains. Nature [Internet] 505:43–49. Available from: http://europepmc.org/abstract/med/24352235

Puga A, Xia Y, Elferink C. 2002. Role of the aryl hydrocarbon receptor in cell cycle regulation. Chem. Biol. Interact. [Internet] 141:117–130. Available from: http://www.sciencedirect.com/science/article/pii/S0009279702000698

Racimo F. 2016. Testing for Ancient Selection Using Cross-population Allele Frequency Differentiation. Genetics [Internet] 202:733 LP-750. Available from: http://www.genetics.org/content/202/2/733.abstract

Racimo F, Kuhlwilm M, Slatkin M. 2014. A test for ancient selective sweeps and an application to candidate sites in modern humans. Mol. Biol. Evol. [Internet] 31:3344–3358. Available from: http://europepmc.org/abstract/med/25172957

Razafsky D, Ward C, Potter C, Zhu W, Xue Y, Kefalov VJ, Fong LG, Young SG, Hodzic D. 2016. Lamin B1 and lamin B2 are long-lived proteins with distinct functions in retinal development. Mol. Biol. Cell [Internet] 27:1928–1937. Available from: http://www.molbiolcell.org/content/27/12/1928.abstract

Ritchie K, Watson LA, Davidson B, Jiang Y, Bérubé NG. 2014. ATRX is required for maintenance of the neuroprogenitor cell pool in the embryonic mouse brain. Biol. Open [Internet] 3:1158 LP-1163. Available from: http://bio.biologists.org/content/3/12/1158.abstract

Rusin SF, Schlosser KA, Adamo ME, Kettenbach AN. 2015. Quantitative phosphoproteomics reveals new roles for the protein phosphatase PP6 in mitotic cells. Sci. Signal. [Internet] 8:rs12 LP-rs12. Available from: http://stke.sciencemag.org/content/8/398/rs12.abstract

Ryan TA. 2006. A pre-synaptic to-do list for coupling exocytosis to endocytosis. Curr. Opin. Cell Biol. [Internet] 18:416–421. Available from: http://www.sciencedirect.com/science/article/pii/S0955067406000895

Ryu EJ, Yang M, Gustin JA, Chang L-W, Freimuth RR, Nagarajan R, Milbrandt J. 2008. Analysis of Peripheral Nerve Expression Profiles Identifies a Novel Myelin Glycoprotein, MP11. J. Neurosci. [Internet] 28:7563 LP-7573. Available from: http://www.jneurosci.org/content/28/30/7563.abstract

Safran M, Dalah I, Alexander J, Rosen N, Iny Stein T, Shmoish M, Nativ N, Bahir I, Doniger T, Krug H, et al. 2010. GeneCards Version 3: the human gene integrator. Database [Internet] 2010:baq020-baq020. Available from: http://dx.doi.org/10.1093/database/baq020

Santarelli S, Wagner K V, Labermaier C, Uribe A, Dournes C, Balsevich G, Hartmann J, Masana M, Holsboer F, Chen A, et al. 2016. SLC6A15, a novel stress vulnerability candidate, modulates anxiety and depressive-like behavior:

involvement of the glutamatergic system. Stress [Internet] 19:83–90. Available from: https://doi.org/10.3109/10253890.2015.1105211

Sekiguchi M, Katayama S, Hatano N, Shigeri Y, Sueyoshi N, Kameshita I. 2013. Identification of amphiphysin 1 as an endogenous substrate for CDKL5, a protein kinase associated with X-linked neurodevelopmental disorder. Arch. Biochem. Biophys. [Internet] 535:257–267. Available from: http://www.sciencedirect.com/science/article/pii/S0003986113001483

Skeide MA, Friederici AD. 2016. The ontogeny of the cortical language network. Nat. Rev. Neurosci. [Internet] 17:323. Available from: http://dx.doi.org/10.1038/nrn.2016.23

Somel M, Rohlfs R, Liu X. 2014. Transcriptomic insights into human brain evolution: acceleration, neutrality, heterochrony. Curr. Opin. Genet. Dev. [Internet] 29:110–119. Available from: http://www.sciencedirect.com/science/article/pii/S0959437X14000963

Sreedharan S, Almén MS, Carlini VP, Haitina T, Stephansson O, Sommer WH, Heilig M, de Barioglio SR, Fredriksson R, Schiöth HB. 2011. The G protein coupled receptor Gpr153 shares common evolutionary origin with Gpr162 and is highly expressed in central regions including the thalamus, cerebellum and the arcuate nucleus. FEBS J. [Internet] 278:4881–4894. Available from: http://dx.doi.org/10.1111/j.1742-4658.2011.08388.x

St-Denis N, Gupta GD, Lin ZY, Gonzalez-Badillo B, Veri AO, Knight JDR, Rajendran D, Couzens AL, Currie KW, Tkach JM, et al. 2017. Phenotypic and Interaction Profiling of the Human Phosphatases Identifies Diverse Mitotic Regulators. Cell Rep. [Internet] 17:2488–2501. Available from: http://dx.doi.org/10.1016/j.celrep.2016.10.078

Stahl R, Walcher T, De Juan Romero C, Pilz GA, Cappello S, Irmler M, Sanz-Aquela JM, Beckers J, Blum R, Borrell V, et al. 2013. Trnp1 Regulates Expansion and Folding of the Mammalian Cerebral Cortex by Control of Radial Glial Fate. Cell [Internet] 153:535–549. Available from: http://dx.doi.org/10.1016/j.cell.2013.03.027

Stocco dos Santos RC, Castro NHC, Lillia Holmes A, Beçak W, Tackels-Horne D, Lindsey CJ, Lubs HA, Stevenson RE, Schwartz CE. 2003. Stocco dos Santos X-linked mental retardation syndrome: Clinical elucidation and localization to Xp11.3–Xq21.3. Am. J. Med. Genet. [Internet] 118A:255–259. Available from: http://doi.wiley.com/10.1002/ajmg.a.20021

Suzuki R, Ferris HA, Chee MJ, Maratos-Flier E, Kahn CR. 2013. Reduction of the Cholesterol Sensor SCAP in the Brains of Mice Causes Impaired Synaptic Transmission and Altered Cognitive Function. PLOS Biol. [Internet] 11:e1001532. Available from: https://doi.org/10.1371/journal.pbio.1001532

Takata A, Miyake N, Tsurusaki Y, Fukai R, Miyatake S, Koshimizu E, Kushima I, Okada T, Morikawa M, Uno Y, et al. 2018. Integrative Analyses of De Novo Mutations Provide Deeper Biological Insights into Autism Spectrum Disorder. Cell Rep. [Internet] 22:734–747. Available from: http://dx.doi.org/10.1016/j.celrep.2017.12.074

Tarpey PS, Raymond FL, O'Meara S, Edkins S, Teague J, Butler A, Dicks E, Stevens C, Tofts C, Avis T, et al. 2007. Mutations in CUL4B, Which Encodes a Ubiquitin E3 Ligase Subunit, Cause an X-linked Mental Retardation Syndrome Associated with Aggressive Outbursts, Seizures, Relative Macrocephaly, Central Obesity, Hypogonadism, Pes Cavus, and Tremor. Am. J. Hum. Genet. [Internet] 80:345–352. Available from: http://dx.doi.org/10.1086/511134

Theofanopoulou C, Gastaldon S, O'Rourke T, Samuels BD, Messner A, Martins PT, Delogu F, Alamri S, Boeckx C. 2017. Self-domestication in Homo sapiens: Insights from comparative genomics. PLoS One [Internet] 12:e0185306. Available from: https://doi.org/10.1371/journal.pone.0185306

Thiru P, Kern DM, McKinley KL, Monda JK, Rago F, Su K-C, Tsinman T, Yarar D, Bell GW, Cheeseman IM. 2014. Kinetochore genes are coordinately up-regulated in human tumors as part of a FoxM1-related cell division program. Mol. Biol. Cell [Internet] 25:1983–1994. Available from: http://www.molbiolcell.org/content/25/13/1983.abstract

Trazzi S, Fuchs C, Viggiano R, De Franceschi M, Valli E, Jedynak P, Hansen FK, Perini G, Rimondini R, Kurz T, et al. 2016. HDAC4: a key factor underlying brain developmental alterations in CDKL5 disorder. Hum. Mol. Genet. [Internet] 25:3887–3907. Available from: http://dx.doi.org/10.1093/hmg/ddw231

Tungadi EA, Ito A, Kiyomitsu T, Goshima G. 2017. Human microcephaly ASPM protein is a spindle pole-focusing factor that functions redundantly with CDK5RAP2. J. Cell Sci. [Internet] 130:3676 LP-3684. Available from: http://jcs.biologists.org/content/130/21/3676.abstract

Vargha-Khadem F, Gadian DG, Copp A, Mishkin M. 2005. FOXP2 and the neuroanatomy of speech and language. Nat. Rev. Neurosci. [Internet] 6:131. Available from: http://dx.doi.org/10.1038/nrn1605

Verheijen MHG, Camargo N, Verdier V, Nadra K, de Preux Charles A-S, Médard J-J, Luoma A, Crowther M, Inouye H, Shimano H, et al. 2009. SCAP is required for timely and proper myelin membrane synthesis. Proc. Natl. Acad. Sci. [Internet] 106:21383–21388. Available from: http://www.pnas.org/content/106/50/21383.abstract

Vernes SC, Oliver PL, Spiteri E, Lockstone HE, Puliyadi R, Taylor JM, Ho J, Mombereau C, Brewer A, Lowy E, et al. 2011. Foxp2 Regulates Gene Networks Implicated in Neurite Outgrowth in the Developing Brain. PLOS Genet. [Internet] 7:1–17. Available from: https://doi.org/10.1371/journal.pgen.1002145

Vernot B, Tucci S, Kelso J, Schraiber JG, Wolf AB, Gittelman RM, Dannemann M, Grote S, McCoy RC, Norton H, et al. 2016. Excavating Neandertal and Denisovan DNA from the genomes of Melanesian individuals. Science (80-.). [Internet] 352:235–239. Available from: http://science.sciencemag.org/content/352/6282/235.abstract

El Waly B, Buhler E, Haddad M-R, Villard L. 2015. Nhej1 Deficiency Causes Abnormal Development of the Cerebral Cortex. Mol. Neurobiol. [Internet] 52:771–782. Available from: https://doi.org/10.1007/s12035-014-8919-y

Wang C, Liang C-C, Bian ZC, Zhu Y, Guan J-L. 2013. FIP200 is required for maintenance and differentiation of postnatal neural stem cells. Nat. Neurosci. [Internet] 16:532. Available from: http://dx.doi.org/10.1038/nn.3365

Weyer S, Pääbo S. 2016. Functional Analyses of Transcription Factor Binding Sites that Differ between Present-Day and Archaic Humans. Mol. Biol. Evol. [Internet] 33:316–322. Available from: http://dx.doi.org/10.1093/molbev/msv215

Widagdo J, Fang H, Jang SE, Anggono V. 2016. PACSIN1 regulates the dynamics of AMPA receptor trafficking. Sci. Rep. [Internet] 6:31070. Available from: http://dx.doi.org/10.1038/srep31070

Wojcik MH, Okada K, Prabhu SP, Nowakowski DW, Ramsey K, Balak C, Rangasamy S, Brownstein CA, Schmitz-Abe K, Cohen JS, et al. 2018. De novo variant in KIF26B is associated with pontocerebellar hypoplasia with infantile spinal muscular atrophy. Am. J. Med. Genet. Part A [Internet] 0. Available from: https://doi.org/10.1002/ajmg.a.40493

Yap CC, Vakulenko M, Kruczek K, Motamedi B, Digilio L, Liu JS, Winckler B. 2012. Doublecortin (DCX) Mediates Endocytosis of Neurofascin Independently of Microtubule Binding. J. Neurosci. [Internet] 32:7439 LP-7453. Available from: http://www.jneurosci.org/content/32/22/7439.abstract

Yigit G, Wieczorek D, Bögershausen N, Beleggia F, Möller-Hartmann C, Altmüller J, Thiele H, Nürnberg P, Wollnik B. 2016. A syndrome of microcephaly, short stature, polysyndactyly, and dental anomalies caused by a homozygous KATNB1 mutation. Am. J. Med. Genet. Part A [Internet] 170:728–733. Available from: http://dx.doi.org/10.1002/ajmg.a.37484

Yoder M, Hildebrand JD. 2007. Shroom4 (Kiaa1202) is an actin-associated protein implicated in cytoskeletal organization. Cell Motil. Cytoskeleton [Internet] 64:49–63. Available from: http://doi.wiley.com/10.1002/cm.20167

Yoshida MM, Azuma Y. 2016. Mechanisms behind Topoisomerase II SUMOylation in chromosome segregation. Cell Cycle [Internet] 15:3151–3152. Available from: https://doi.org/10.1080/15384101.2016.1216928

Zachos G, Black EJ, Walker M, Scott MT, Vagnarelli P, Earnshaw WC, Gillespie DAF. 2017. Chk1 Is Required for Spindle Checkpoint Function. Dev. Cell [Internet] 12:247–260. Available from: http://dx.doi.org/10.1016/j.devcel.2007.01.003

Zeng K, Bastos RN, Barr FA, Gruneberg U. 2010. Protein phosphatase 6 regulates mitotic spindle formation by controlling the T-loop phosphorylation state of Aurora A bound to its activator TPX2. J. Cell Biol. [Internet] 191:1315 LP-1332. Available from: http://jcb.rupress.org/content/191/7/1315.abstract

Zhou H, Hu S, Matveev R, Yu Q, Li J, Khaitovich P, Jin L, Lachmann M, Stoneking M, Fu Q, et al. 2015. A Chronological Atlas of Natural Selection in the Human Genome during the Past Half-million Years. bioRxiv [Internet]. Available from: http://biorxiv.org/content/early/2015/06/19/018929.abstract
